# “Gut” to grips with the science of the microbiome – a symposium report

**DOI:** 10.1017/gmb.2024.8

**Published:** 2024-11-29

**Authors:** Yvonne E. Finnegan, Holly R. Neill, Emily J. Prpa, Bruno Pot

**Affiliations:** 1 Yvonne Finnegan FINNE Nutrition & Regulatory Consultancy, Kilkenny, Ireland; 2 Yakult UK & Ireland Ltd, London, UK; 3 Yakult Europe BV, Science Department, Almere, The Netherlands

**Keywords:** probiotic, prebiotic, fermented foods, gut microbiome, gut microbiota

## Abstract

The latest Yakult Science Study Day was held virtually on 2 November 2023. Aimed at healthcare professionals, researchers, and students, a variety of experts explored the latest gut microbiome research and what it means in practice. The morning sessions discussed the role of the microbiome in health and disease, the rapid advancements in DNA sequencing and implications for personalised nutrition, the current state of evidence on health benefits associated with fermented foods, prebiotics and probiotics and the challenges involved in interpreting research in this area. The afternoon session considered the emerging research on the microbiota–gut–brain axis in mediating effects of food on mood, the bidirectional impact of menopause on the gut microbiota, and the interplay between the gut and skin with implications for the treatment of rare and common skin disorders. The session ended with an update on the use of faecal microbiota transplant in both research and clinical practice. Undoubtedly, the gut microbiome is emerging as a key conductor of human health, both in relation to gastrointestinal and non-gastrointestinal outcomes. As research continues to elucidate mechanisms of action and confirm their effects in human trials, the gut microbiome should be a key consideration within a holistic approach to health moving forward.

## Introduction

The past decade has seen an exponential growth in research publications on the gut microbiome in recognition of the fact that the trillions of microorganisms that are present in the gut interact actively with the host. These interactions involve not only digestive processes but also immune, metabolic, neural, and endocrine pathways (Kasarello et al., 2023) and have triggered a shift away from simply cataloguing organisms towards a greater understanding of their functions, interactions, and impacts on health and disease. Nowadays, the gut microbiome is being considered as the conductor in the orchestra of human health. While unmodifiable factors such as age and genetics impact our microbiome, many lifestyle factors (e.g., diet, physical activity, stress, sleep) are amenable to change and offer the potential for a more holistic approach to health. The microbiome is also being explored within healthcare in areas of disease diagnosis (Gulliver et al., 2022), the development or optimisation of medicines (O’Reilly et al., 2023) and as a possible adjunct therapy (Sarris et al., 2022). Exploration of microbiomes found in other parts of the body, including the skin and vagina, and their roles in clinical conditions are also further emerging (Aggarwal et al., 2023).

Parallel to the increasing numbers of scientific publications, there has been a surge in public interest in gut health and diet as evidenced in media reports and online contributions in this area. It is therefore important that practitioners are up-to-date with recent scientific advances as well as the limitations of the current research in order to appropriately inform those seeking advice relating to gut health. With this in mind, the latest Yakult Science Study Day was held online on 2 November 2023, hosted by Yakult Science for Health UK & Ireland in partnership with MyNutriWeb. The study was approved for continual professional development by the Association for Nutrition and content endorsed by the Gastroenterology specialist group of the British Dietetic Association. This report summarises talks from leading experts on the current state of evidence as well as recent advances in the field of microbiome research and what this means for clinical practice.

## Gut microbiota and personalised nutrition – a focus on fermented foods

Next-generation sequencing techniques have helped advance our understanding of both the human microbiome and the complex microbial communities found in fermented foods and offer the potential for a more personalised approach towards optimal nutrition in the future. This was the topic discussed by Professor Paul Cotter, Head of Food Biosciences at Teagasc Food Research Centre, principal investigator of the APC Microbiome and VistaMilk Centres in Ireland and the CTO of SeqBiome.

### Microbiome research – from the tip of the iceberg to the full glacier

Although often used interchangeably, differences exist between the terms “microbiota” and “microbiome.” “Microbiota” describes the microorganisms that are found in a defined environment, while the term “microbiome” is broader and refers to the collection of genomes from all the microorganisms in the environment, including the microorganisms themselves as well as microbial structural elements, metabolites, and the environmental conditions (Berg et al., 2020).

As many microbes do not grow on standard media, traditional agar or culture-based approaches in microbiology provide scientists with only a fraction, or the “tip of the iceberg,” of the potential picture of the microbiome present in a particular environment. The emergence of culture-independent techniques, such as DNA sequencing, allows the microorganisms present to be more fully investigated based on the DNA found in any sample, giving information about microbes that are difficult to grow or currently cannot be grown in the laboratory. Notably, the sample being prepared for analysis must be of good quality and collected in a manner to ensure that it is representative of the DNA from the environment under investigation.

Two main types of DNA sequencing are used to study the microbiome – amplicon sequencing and shotgun metagenomics. Amplicon sequencing techniques mostly focuses on the 16S rRNA sequences present in all bacteria. In the case of fungi, Internal Transcribed Spacer sequences are used. The 16S rRNA gene contains conserved DNA regions easily found by a universal PCR primer. Between these conserved regions, bacteria have highly variable sequences, which are then amplified and can be used to identify the taxonomic status of bacteria using reference databases. This technique facilitates identification of the microorganisms down to the genus level (e.g., *Lactobacillus* or *Bifidobacterium*) but rarely at the species level. This might be important as functional benefits have been shown to be species-specific or even strain-specific.

For this reason, shotgun metagenomics, allowing identification at species and strain levels, has become more popular. Shotgun metagenomic-based approaches sequence all the genes present in a community, including genes from bacteria, yeasts, fungi, viruses, phages, and small eukaryotes. In addition to identifying the members present in the microbial communities, shotgun metagenomics also provides information about the pathways encoded within those microorganisms. This yields valuable information about the functional potential of the ecosystem; for example, it can also provide an assessment of the number of genes associated with health-promoting attributes or undesirable traits (e.g., virulence factor, antibiotic resistance genes). The identification at the strain level is key for probiotic research because the majority of probiotic traits are strain-specific. Shotgun sequencing allows researchers to assemble the entire genome of a microorganism by creating the so-called metagenome assembled genomes, even if the microorganism has never been grown in a laboratory. Based on the genome sequences, it is possible to predict metabolic pathways and whether the organism is likely to be health promoting or not. DNA sequencers have evolved considerably in the last 4–5 years. Overall, the quality of the sequencing has improved, as well as the length of the sequence fragments, leading to more reliable assemblies of more complete genomes.

### Why does microbial diversity matter?

DNA sequence analysis also allows an assessment of the microbial diversity of a sample. Microbial diversity appears to be critical in maintaining resilience, which can be defined as the speed or extent to which the microbial ecosystem will recover its initial function or composition following perturbations (Fassarella et al., 2021). Using an ecological analogy, the large number of species present in a rainforest makes it more resilient to the loss of a few species, for example, due to predators or severe weather events. The reason is that the functions of the lost species may more likely be replaced by other species in that environment, allowing the complete ecosystem to continue to thrive. Conversely, in a desert environment with less species diversity, such an event has more serious implications, as there is less capability to replace the functional activities of the lost species. Similarly, a gut microbiota that is more diverse before exposure to stressors such as infection, antibiotic use, stress, or illness may resist better against major changes that can impact host health. Diet may play a key role in developing a diverse and resilient gut microbiota (Fassarella et al., 2021).

Alpha and beta diversity refer to the species richness (number) or distribution (evenness) *within* a community and the difference in the number of species *between* different ecosystems, respectively (Andermann et al., 2022). For example, a study of microbial diversity in a selection of fermented foods detected 301 different microbial species in brine, 242 in sugar, and 70 in dairy foods (Leech et al., 2020). Beta diversity analysis highlighted that these microbiomes clustered distinctly reflecting the substrates (i.e., brine, sugar and dairy) used to produce these foods. It is important to note that species numbers should not be the only measure of diversity in microbiome research but should preferably also include gene diversity, functional potential, and metabolic capability of the microbiota.

### Prebiotics to enrich next generation health-promoting microbes – a personalised approach?

*Human milk oligosaccharides:* There is continued interest in more personalised approaches in relation to interventions that may improve health through microbiome-targeted activities. A prebiotic group that has gained a lot of interest in this area is the human milk oligosaccharides (HMOs). These are complex multifunctional glycans are present in human breast milk at different levels and compositions, depending on the mother’s genetic background and diet. They are resistant to digestion in the small intestine and reach the infant’s colon in an intact form where they are metabolised by some gut microorganisms. Over 150 HMO structures have been identified with different functionality, sparking increased interest in their use beyond infant formula. Particular HMOs have the potential to dictate microbiota composition that may find application for personalised nutrition whereby a particular HMO may be used to target an increase in a specific *Bifidobacterium* that may be deficient in an individual submitted but more research is required (Lordan et al., 2024).

Beyond *Lactobacillus and Bifidobacterium:* There are many additional desirable microorganisms beyond species from the genera *Lactobacillus* and *Bifidobacterium* that are associated with health benefits, such as *Akkermansia muciniphila, Ruminococcus bromii, Faecalibacterium prausnitzii*, and *Roseburia intestinalis. A. muciniphila* is associated with a lean phenotype, whereas *F. prausnitzii* has been found to be present at high levels in individuals without inflammatory bowel disease (IBD). By extension, the absence of this microorganism in IBD suggests that encouraging its growth might contribute to alleviating some of the associated symptoms (Lordan et al., 2020). However, challenges can exist when incorporating these microorganisms into foods as they are extremely sensitive to oxygen, difficult to grow in sufficient amounts, or have not (yet) been sufficiently characterised for safety and functionality to allow them to be used in a dietary supplement. A strategy to maintain high levels of these microorganisms is to target them through dedicated dietary interventions that encourage their growth as summarised in a recent publication (Lordan et al., 2020).

New opportunities for personalised nutrition may also come from metabolic modelling tools that can be used to predict which nutrients will support the growth of microorganisms that have never previously been cultured (Machado et al., 2018). This “cocktail of ingredients” could contain a particular prebiotic, vitamin, mineral, or carbohydrate source that confers a selective advantage on that microorganism, providing the basis for a more personalised or targeted intervention. An alternative route to personalised nutrition is to identify a specific goal for an individual, such as an increase in the levels of desirable metabolites, such as short-chain fatty acids (SCFAs; acetate, butyrate and propionate), vitamins, or antioxidants, and target interventions that address this goal (Lordan et al., 2024).

### Focus on fermented foods

Fermented foods have been defined as “foods that are made through the desired microbial growth and enzymatic conversion of food components” (Marco et al., 2021). These foods have generally been consumed for thousands of years (e.g., beer, wine, cheese, fermented meat, kefir, kimchi, etc.) and the microorganisms present dictate the outcome of the fermentation process. The original purpose of fermentation was to preserve food, but over time, knowledge has grown around their potential health benefits, as well as their ability to enhance texture and flavour of the final product. Arguments have been made to suggest that the move away from fermented foods in Western societies over the last century, coupled with many other factors such as increased hygiene and sterility of our food and environments, reduced rates of breastfeeding, and overuse of antibiotics, has contributed to the increase in non-communicable diseases (Sonnenburg et al., 2016).

Consumption of fermented foods has been shown to positively influence the gut microbiota and could exert benefits through a number of potential pathways, including the delivery of live microorganisms to the gut, modulating the gut microbiota (Marco et al., 2021; Mukherjee et al., 2024). A large-scale meta-analysis comparing the overlap of lactic acid bacteria (LAB) from food sources and human body sites, demonstrated a close relation between the two, providing increasing evidence that fermented foods can be a possible source of LAB for the gut microbiota (Pasolli et al., 2020).

Depending on the strains present, fermented foods can alter the nutritive qualities of the food through activities such as pre-digestion (e.g., lactose), elimination of toxins (e.g., phytic acid) or production of vitamins. Microbial-derived products made during fermentation, for example, peptides, bacteriocins, and amino acids, may also exert benefits by changing intestinal or systemic function. In foods that have been pasteurised or baked, thus killing any live microbes (e.g., sourdough bread), beneficial attributes may still exist depending on the presence of microbial metabolites and the mechanism of action associated with it. While Professor Cotter cautions against heeding advice from some influencers citing fermented foods as a “cure-all,” he believes that encouraging daily consumption of fermented foods is a useful way to introduce more microbes in our diet.

### Not all fermented foods are created equally

It is important to clearly differentiate between fermented foods and probiotics. The term “probiotic” is retained for a strain-specific microorganism that has been studied in-depth and for which a particular health effect has been established through well conducted clinical trials (see the following presentation by Professor Pot). For many fermented foods, evidence is lacking regarding the specific health benefits of the microbes present, and the exact quantities of microbes remain often undefined. A greater understanding of the health effects of fermented foods based on high-quality data available from population-based diet studies and randomised controlled trials (RCTs) are needed. Until such evidence is available, and depending on the regulatory environment also, such products should not be labelled as “containing probiotics” unless a specific probiotic strain has been added.

Kefir, a fermented milk beverage, has been consumed for thousands of years. Significant differences can be found in the microbial composition of individual samples of kefir. Many commercial examples of kefir do not typically contain acetic acid bacteria, which are often present in traditional kefirs, or contain *Lactobacillus* species that are different from those found in traditional kefir or kefir grains. Traditional kefirs also tend to have more complex yeast communities compared to commercial products (Bourrie et al., 2021).

These differences can impact not only on the final flavour of the fermented product but also on the potential health benefits as demonstrated in a recent RCT in 21 adult males fed a commercial versus a traditional kefir (350 g twice daily for a period of 4 weeks). Compared to baseline, traditional kefir consumption reduced the levels of both LDL cholesterol (LDL-C) and the intercellular adhesion molecule-1 and vascular cell adhesion molecule-1 (VCAM-1), while commercial kefir consumption increased tumour necrosis factor-α (TNF-α). The traditional kefir resulted in greater reductions in Interleukin-8 (IL-8), C-reactive protein, VCAM-1, and TNF-α when compared to commercial kefir consumption (Bourrie et al., 2023). This work illustrates the importance of identifying the specific microbial composition when linking fermented foods to health benefits.

## Key messages

- New DNA sequencing techniques are revolutionising our understanding of the gut microbiome, leading to the identification of newer health-promoting species and the prediction of their metabolic pathways that can help determine how to more specifically promote their growth.
- Opportunities to personalise interventions to target the microbiome are at early stages but could target not only microbiota diversity but also changes in specific metabolites known to have beneficial effects, such as SCFAs.
- A large variety of fermented foods are consumed around the world and have a long history of safe use. However, health benefits depend on the specific microbial community present, and few have been subjected to the type of clinical research trials needed to prove these benefits. Future research should focus on standardisation and quality control around specific strains to maximise the potential health benefits of fermented foods.

## Probiotics and the gut microbiota: key players in health?

How the gut microbiota can be influenced by probiotics, their impact on health and the challenges involved in researching this area was discussed by Professor Bruno Pot, Science Director at Yakult Europe B.V and lecturer at the Free University of Brussels.

Probiotics are defined as “Live microorganisms that, when administered in adequate amounts, confer a health benefit on the host” (Hill et al., 2014). The definition means that in principle, probiotics are not limited to bacteria but can include yeast, archaea, and fungi and could be taken as foods, supplements, or even drugs. It excludes indigenous microorganisms (from contact with skin or from the oral cavity) and dead and heat-killed cultures. Crucial in the definition is that a probiotic should be well characterised (e.g., at strain level), alive at the time of consumption and the health effects are supported by good quality clinical studies.

The list of possible health benefits for probiotics is growing steadily and may have reached a point where their credibility is being called into question. However, the past decade of research has revealed the role of the microbiota in aspects of health beyond the gut from obesity and allergies to brain and neural disorders. As probiotics can modulate the composition of the gut microbiota – and the diversity of potential strains offers different mechanisms of action – it is reasonable to understand that a broad diversity of probiotic applications is possible.

### How do “good” bacteria influence the immune response?

As we consume approximately 65 tonnes of non-sterile food and drink over our lifetime, it is not unexpected that a great proportion (70%) of our immune system is situated in the gut – where it functions to filter out viruses, toxins and allergens. Pathogenic organisms have the ability to penetrate through the epithelium into the inner part of the body, where they are detected by dendritic cells and are destroyed by the macrophages of the immune system. In this process, immune cells send out signals which, in the case of an inflammatory response to a pathogen, will be characterised by the cytokine IL-12 (Preidis and Versalovic, 2009). IL-12 activates other immune cells (e.g., T-cells) to launch a cascade of inflammatory responses to fight the pathogen. This includes the activation of B lymphocytes which will secrete immunoglobulin A into the lumen of the gut where the pathogen is destroyed and excreted from the body.

Unlike pathogens, commensals (generally beneficial organisms that help maintain a healthy host environment) do not translocate through the gut epithelium, and when detected in the lumen by dendritic cells, they trigger production of the anti-inflammatory Interleuking-10 (IL-10) (Preidis and Versalovic, 2009). The balance of pro- and anti-inflammatory cytokines is important not only in health but also in the control of allergies or auto-immune diseases when the immune system is overactive and inflammation results in damage. Thus, supporting the growth of beneficial bacteria through prebiotics or introducing certain probiotics may provide a means to influence the inflammatory response.

### Multiple mechanisms, multiple responses, and multiple strains – a challenge to research

**Figure 1.**
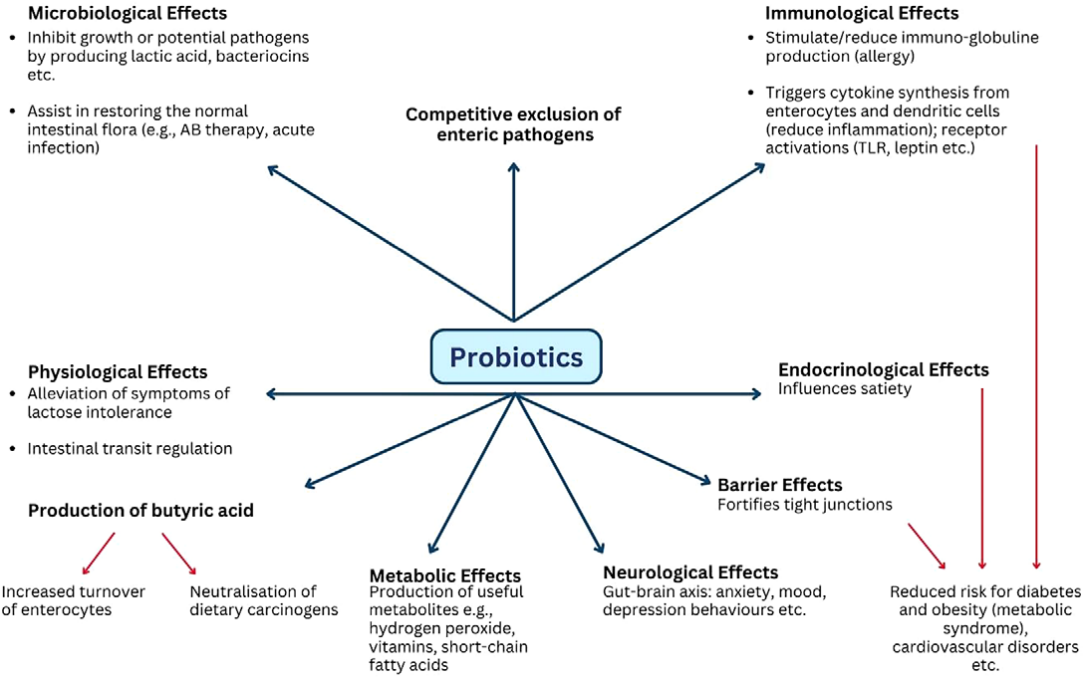
Examples of potential mechanisms observed for probiotics. The application range of probiotic organisms is linked to a wide variety of possible mechanisms. At any given time, more than one mechanism may be active. Several mechanisms may be required simultaneously to achieve a specific beneficial health effect.

To understand the challenges posed in probiotic research, it is important to look at some of the complexities faced. First, it is likely that a probiotic may exert its effects through several mechanisms that may be employed simultaneously and can be either direct effects or mediated indirectly (see Figure 1). The mechanisms include direct competition with pathogens for nutrients and place on the epithelium, production of useful metabolites such as vitamins, SCFAs, or bacteriocins, barrier effects (e.g., fortifying tight junctions), as well as immunological effects and has been summarised more extensively in Chen et al. (2023). For example, a probiotic that produces SCFAs may at the same time also produce growth factors which will indirectly stimulate the growth of other beneficial organisms. For this reason, a careful definition of the endpoints and whether they are immunological or metabolic in nature is needed in probiotic clinical trials. With this endpoint in mind, specific strains can then be selected in the laboratory that most likely will have the best performance in a targeted activity or to obtain a particular outcome (Sánchez et al., 2017).

Second, research shows that it is not sufficient to characterise probiotic effects based on similarities at the genus or species level. *In vitro* research investigating the potential of various species and strains of lactobacilli to induce different levels of the anti-inflammatory cytokine IL-10 or the pro-inflammatory cytokine IL-12 on human peripheral blood mononuclear cells was presented (Foligne et al., 2007). It showed that it was possible to clearly discriminate between strains that have overall anti-inflammatory potential versus those which do not, even within the same species of bacteria, such as *Lacticaseibacillus (Lc.) casei* (Alard et al., 2018). Owing to this strain specificity, probiotic products should be labelled with the exact same strain number as used for the clinical studies and healthcare professionals are advised, when recommending a particular probiotic product, to confirm that the strain used in the product indeed matches the strain used in the supporting clinical research.

Third, variation in individual response is observed in many clinical drug trials, and the same potential exists in the host responses to probiotics. Using the same strain, individuals demonstrate variable capacity to induce the key cytokines IL-10 and IL-12. People with a high potential to produce IL-10, however, will generally show a low capacity to induce IL-12 (Pot et al., 2013; Alard et al., 2018). This observation highlights the need for clinical trials performed on a sufficiently diverse population to accurately represent these variants and ensure that the probiotic illustrates an overall efficacy.

### Systematic reviews and meta-analysis in probiotic research – proceed with caution?

Although systematic reviews and meta-analyses of RCTs are considered the “gold standard” for providing evidence of an *in vivo* effect, they should be approached with caution in the field of probiotic research, due to the heterogeneity of the data in terms of factors such as strain identity, dose and mode of administration used, as well as target population, endpoint variation, and season of investigation.

When closely inspecting meta-analyses in this area, it is clear that the performance of a strain significantly depends on the overall product characteristics, including the food matrix, and whether it is presented as a single strain or in a combination of strains. For example, a meta-analysis of studies investigating probiotics for antibiotic-associated diarrhoea showed a difference in efficacy when a particular probiotic strain was combined with other strains (Agamennone et al., 2018). When *Lacticaseibacillus rhamnosus* GG was given as a single strain, the study results favoured the probiotic. When combined in another study with *Lactobacillus acidophilus* and *Bifidobacterium (B.) animalis* subsp*. lactis* strains, the performance of the multi-strain probiotic was improved. However, in a third study using *Lc. rhamnosus* GG but with a different combination of other strains (including another strain of *Lc. rhamnosus*), the effect favoured the placebo. In addition, results differed when the probiotic was given as a dairy product or as a food supplement (Agamennone et al., 2018).

The quality of the study should also be considered. In the same meta-analysis, two studies showed a neutral effect of a probiotic on antibiotic associated diarrhoea. In one study, the conclusion is based on one diarrhoea case in 49 subjects following probiotic or placebo intervention, while the other was based on 159 cases of antibiotic associated diarrhoea in 14170 subjects receiving the probiotic and 153 cases of diarrhoea in 14171 participants taking the placebo.

Meta-analysis of probiotics should therefore be handled with care and at present well conducted RCT’s may offer the best route for direct proof of efficacy of a particular strain (Agamennone et al., 2018).

## Key messages

- Probiotics can be key players in health, but effects are strain-specific and may rely on very specific mechanisms.
- Clinical trials of probiotics should be carried out with the specific combination of strains and within the food matrix intended to be used in the final commercial product as these factors can impact on the efficacy of the probiotic.
- Probiotic foods and supplements need to be labelled to strain level.
- Studying probiotics is complex due to a combination of variable factors such as diet, stress, lifestyle as well as individual host interactions and the multiplicity of mechanisms that may be involved.
- Care needs to be taken with interpretation from systematic reviews and meta-analyses due to significant heterogeneity in the data. At present, properly powered and conducted double blind RCT’s of specific probiotic strains offer the best level of evidence for their efficacy.

## Current perspectives on gut health research – a focus on prebiotics

The gut harbours the vast majority of the microbial cells which are modifiable to improve health (Sender et al., 2016). Professor Glenn Gibson from University of Reading explained current perspectives on a healthy microbiota and how prebiotics impact health through the modulation of the microbiota. Together with his colleague Professor Marcel B. Roberfroid, Professor Gibson in fact coined the term “prebiotics” in 1995.

### Can we define a healthy gut microbiota?

Microbial numbers gradually increase along the intestinal tract from the relatively sterile environment of the stomach (<10^3^ CFU/ml) to the large intestine, where the combination of slower transit time (48–72 hours in an adult), along with a favourable pH and a constant supply of nutrients from the diet, result in a dramatic increase in microbial numbers (10^11^–10^12^ CFU/ml) (Sender et al., 2016). The interrelationship between the gut microbiota and the host is usually mutually beneficial, with the host supplying nutrients via dietary intake and the microbiota helping the process of homeostasis by providing resistance to infections and reducing inflammation. Various dietary interventions, including prebiotics, are being investigated as modulators of the gut microbiota in order to improve resilience to these stressors.

As each individual has an entirely unique gut microbiota profile owing to numerous modifiable factors, including diet and physical activity as well as non-modifiable factors, such as age, ethnicity, environment, and genetics (Redondo-Useros et al., 2020), which interrelate and react with each other (Qin et al., 2022), it is not possible to define an optimal gut microbiota composition. Nevertheless, a number of initial concepts important for health can be suggested to characterise a “healthy” gut microbiota. Activities that could be considered of positive value to the host include improvements in barrier function, the production of vitamins by bacteria (vitamin B_12_ and K), stimulation of anti-inflammatory cytokines, inhibitory activities towards gastrointestinal (GI) pathogens, and the production of organic acids (e.g., acetate, propionate, butyrate, succinate and lactate). There are also activities that at present appear neither positive nor negative, such as the formation of gases such as H_2_, CO_2_, and CH_4_ which are relatively inert. Finally, there are certain activities which do not appear to be beneficial to the host, including toxic gas formation (H_2_S), certain products of proteolytic metabolism (phenol, indole, skatole, ammonia, and branched chain fatty acids), and known bacterial toxins from *Shigella* spp., enterotoxigenic *Escherichia coli, Cl. difficile*, and *Clostridium perfringens.* Better understanding of the exact relationship between these various activities may assist in defining and quantifying a “healthy” gut microbiota.

### The prebiotic research landscape – mechanisms and impact on health and disease

Prebiotics are defined as “substrates that are selectively utilised by host microorganisms conferring a health benefit” (Gibson et al., 2017). They work by selective proliferation of beneficial bacteria, which in turn also inhibit pathogenic organisms. Both *in vitro* and *in vivo* methods are used to investigate the mechanisms of action of prebiotics, which share some common features with probiotics (Chen et al., 2023).

Prebiotics include, besides HMOs, readily fermentable dietary fibres, conjugated linoleic acids, polyunsaturated fatty acids, fructo-oligosaccharides (FOS), galacto-oligosaccharides (GOS), mannan-oligosaccharides, xylo-oligosaccharides, and certain phenolic compounds and phytochemicals. However, the levels of evidence for health benefits of these substances vary considerably with the highest weight of evidence and mechanistic understanding for inulin, GOS, and FOS (Gibson et al., 2017).

*In vitro* methods at the University of Reading apply batch culture fermenters for the initial screening of candidate prebiotics using a single pH, transit time, and substrate concentration. Once a candidate prebiotic has been identified, it is studied in a more complex, multi-chambered continuous flow system that replicates the conditions of the proximal, transverse, and distil areas of the colon. This permits prediction of the part of the colon where the prebiotic may exert its influence. A desirable trait for a prebiotic is to target the more distal areas of the gut where the majority of gut diseases are known to originate, for example, colitis, colon cancer. While these models serve a purpose in providing initial information on potential mechanisms of action, the definitive test of a prebiotic is by *in vivo* human intervention studies. The newer molecular microbiology methods for characterising the microbiota metabolism will also help to provide the necessary mechanistic evidence that support effects of prebiotics. A selection of RCTs of prebiotic interventions in humans across a range of health conditions were presented.

In vivo *prebiotic trials in adults*: The early days of prebiotic studies were characterised by feeding prebiotics and measuring the effects on gut microbiota, as exemplified in a trial of healthy participants assigned to consume a novel or commercially available GOS versus placebo (Depeint et al., 2008). Those consuming the GOS showed significant dose-dependent increases in bifidobacteria. Such studies were followed by RCTs that attempted to additionally characterise both the health impact along with changes in the microbiota.

An RCT supplementing overweight adults at risk of metabolic syndrome (n = 45) with a GOS mixture (5.5 g/d) for 12 weeks, resulted in an increase in *Bifidobacterium* and a significant beneficial change in the immunomarkers, plasma C-reactive protein, and faecal calprotectin, compared to placebo. Insulin, total cholesterol, and triglyceride concentrations were also significantly lower at 12 weeks, but weight and blood pressure remained stable (Vulevic et al., 2013). For outcomes such as weight management, it is likely that longer term trials are necessary.

In ageing, there is a marked decrease in the numbers of beneficial bifidobacteria in the gut and a decline in immune function. Supplementing healthy elderly subjects (n = 44) with GOS (5.5 g/d) for 10 weeks resulted in a significant increase in bifidobacteria and phagocytosis, natural killer cell activity, and production of the anti-inflammatory cytokine IL-10 compared with placebo. This demonstrates the potential of prebiotics to attenuate age-related decline in immune function (Vulevic et al., 2008).

Use of the prebiotic FOS has been associated with reduced recurrence of *Cl. difficile*-associated diarrhoea and reduced recovery time from the consequences of antibiotic treatment, accompanied by an increase in faecal *Bifidobacterium* (Lewis et al., 2005). Similarly, GOS has been shown to significantly reduce the incidence and duration of travellers’ diarrhoea compared to placebo (Drakoularakou et al., 2010). These data led to further research into athletes and military personnel who travel frequently and where diarrhoea can have a significant impact on performance.

Prebiotics interventions have also been tested in patients suffering from irritable bowel syndrome (IBS), where a GOS supplement resulted in improvements in stool consistency, flatulence, bloating, and composite score of symptoms, which tallied with an increase in *Bifidobacterium* as compared to placebo (Silk et al., 2009).

In vivo *prebiotic trials in infants:* Infants fed a formula including a combination of short-chain GOS (scGOS) and long-chain FOS (lcFOS) had significantly less cumulative gut and respiratory infections at 18 months versus those fed a standard formula (Ivakhnenko and Nyankovskyy, 2013). Additionally, the prebiotics conferred a similar level of protection to that observed in breastfed infants. In a prospective RCT trial in healthy term infants at risk of atopy, a prebiotic-supplemented formula offered in the first 6 months of life significantly reduced the 2-year cumulative incidences of any allergic manifestation compared to the placebo group (Arslanoglu et al., 2008). Furthermore, the 5-year cumulative incidences of any allergic manifestation and atopic dermatitis (AD) were significantly lower in the scGOS/lcFOS group compared to the placebo group (Arslanoglu et al., 2012).

### Future horizons for prebiotics

Moving forward, it is likely that different prebiotics will be required to promote the growth of the more recently identified probiotic organisms in genera such as *Akkermansia*, *Roseburia*, and *Eubacteria.* Unlike the well-established targets such as *Lactobacillus* and *Bifidobacterium* species, various newly discovered bacteria produce additional SCFAs such as propionic acid and butyric acid, which are also of interest in supporting the gut microbiota. This may lead to more work on synbiotics, which can be either complementary (pre and probiotics acting independently but administered together) or synergistic (pre and probiotics acting together). In addition, different prebiotics will also be needed to target the range of commensals found in areas beyond the GI tract, including the skin, urogenital tract, placenta, vagina, and oral cavity.

Future work should focus on generating data on mechanisms of action to support evidence, while the inclusion of human cells and dialysis techniques in artificial gut models will help improve the replication of real-life gut conditions. Increasing our understanding of the human metabolome (the complete set of metabolites found in a biological sample, e.g., blood, urine) and any relationships with microbial profiles during intervention trials will also assist in the understanding of mechanisms. It is likely that the greatest potential for prebiotics is as a prophylactic treatment in healthy populations or as an adjunct clinical therapy rather than fully replacing pharmaceutical interventions. Greater consideration should be given to recommending prebiotic interventions following the administration of antibiotics to help restore the gut microbiota.

## Key messages

- Prebiotics are defined as “substrates that are selectively utilised by host microorganisms conferring a health benefit.”
- A wide range of substances have been identified that are selectively utilised by host micro-organisms but fewer have the necessary level to support a health benefit from human trials. The prebiotics inulin, GOS and FOS currently demonstrate the highest strength of evidence and mechanistic understanding of health benefits.
- While *in vivo* studies are essential for confirming health benefits in humans, *in vitro* studies are useful for revealing candidate prebiotics, or mode of action.
- The identification of previously unknown probiotic species outside of the gut will require the identification of new or alternative prebiotics which support their growth and function to deliver health benefits.

## Food, mood, and the gut microbiome

Humans may instinctively have felt a connection between the gut and the brain as evidenced by everyday parlance around having a “gut feeling” or the presence of “butterflies in the stomach.” There is growing evidence for a role of diet in mood and mental health, which may be mediated through the interaction between the gut microbiome and the gut–brain axis. This was the topic explored by Dr Emily Leeming, a registered dietician, researcher at King’s College London, and author of *Genius Gut: The Life-changing Science of Eating for Your Second Brain.* Dr Emily Leeming also writes a popular weekly Substack newsletter called *Second Brain* on the gut–brain connection.

### Evidence for a role of diet in mood and depression

Beyond temporary changes in mood arising from the enjoyment of palatable food, there is growing evidence for the role of diet in maintaining positive mood and preventing or decreasing the severity of mood disorders. Observational studies suggest a stepwise increase between happiness and diet quality (Mujcic and J Oswald, 2016). In an RCT, providing participants (n = 75) with their recommended amount of vegetables for 8 weeks improved their diet quality and ratings on the subjective happiness scale versus a placebo group who received no additional vegetables (De Leon et al., 2022).

Depression is a devastating mood disorder and the biggest contributor to disability worldwide. There is a scarcity of data on the pathophysiology of depression, and there is little consistent evidence that depression is caused by a chemical imbalance in the brain, particularly serotonin (Moncrieff et al., 2023). At present, available treatments induce remission in only half of the time (Casacalenda et al., 2002), and adjunct therapy based on modifiable risk factors are being explored.

The role of seven lifestyle factors (alcohol consumption, diet, physical activity, sleep, smoking, sedentary behaviour, and social connection) in depression were investigated in almost 300,000 participants followed up over 9 years (Zhao et al., 2023). The results demonstrated the independent protective roles of certain factors on clinically diagnosed depression, including a healthy diet, which decreased the risk by 6% and was assessed based on adherence to U.S. dietary guidelines.

A meta-analysis of observational studies has shown that a healthy diet and, in particular, a Mediterranean-style diet, was strongly linked to lower rates of depression (Lassale et al., 2019) while diets high in ultra-processed foods were reported to be linked to a 23% higher risk of depression (Lane et al., 2023). However, one of the issues with observational studies is reverse causality; low mood and stress can potentially change eat patterns towards more high-calorie, nutrient-poor “comfort foods” (Firth et al., 2020) whilst feelings of tiredness arising from low mood could contribute to a reduced desire to cook healthy meals. For this reason, RCTs offer more concrete evidence for a link between diet and mood.

A 12-week landmark RCT in Australia (The SMILES Trial) investigated dietary changes using a modified Mediterranean diet in participants (n = 67) suffering from moderate to severe depression whose diets were of poor quality (Jacka et al., 2017). The dietary intervention comprised personalised dietary advice and nutritional counselling from a clinical dietician to support optimal adherence to the recommended diet. The placebo group did not receive any dietary advice but was provided with the same schedule and length of social visits as the intervention group. The diet group demonstrated significantly greater improvement in ratings of depression using the Montgomery–Åsberg Depression Rating Scale versus the placebo group. Remission from depression was achieved for 32.3% of the diet group and only 8.0% of the social support group. Similar findings were replicated in another 12-week RCT in young men suffering from depression who followed a Mediterranean diet intervention. The diet led to a significant reduction in depression (as measured by the Beck Depression Inventory) versus the control group who only received social support (Bayes et al., 2022).

In summary, a combination of observational and RCTs have demonstrated the potential impact of diet on mood and mental health, which may be mediated by many factors, including the effects of nutrients on function and brain structure and also potentially through modulation of the gut microbiome.

### The gut as a “chatty” neighbour to the brain

The brain is an organ that is still in discovery, as shown by the recent publication of the “largest atlas of human brain cells,” revealing more than 3,000 types of cells, many of which were newly discovered (Maroso, 2023). The brain is regarded as the “control centre” and communicates with many organs, including the gut, via the vagus nerve. However, evidence of a bidirectional relationship between the brain and gut has existed for some time, through knowledge around hunger and satiety signalling from the gut to the brain and conversely the impact of mental stress (triggering the flight or fight response) on gut symptoms. What is not often appreciated, however, is that 80% of the neurons in the vagus nerve are sensory afferent neurons conveying information “up” from the intestinal wall to the brain, informing the brain around the status of the gut and dietary status (Breit et al., 2018).

The reference to the gut being the “second brain” refers to the enteric nervous system (ENS); a network of around 500 million neurons embedded along the GI tract that connects to the vagus nerve (Fleming et al., 2020). The ENS autonomously controls digestion and can communicate with the brain via several neuromodulators and neurotransmitters, including epinephrine, norepinephrine, serotonin, dopamine, γ-aminobutyric acid, and acetylcholine as well as via microbial metabolites, such as SCFAs (Montagnani et al., 2023). It is therefore possible that any disturbance or dysregulation of either gut or brain pathways can potentially affect the functionality of the other.

### Microbiota–gut–brain axis: emerging evidence

Although it has been recognised that there is a bidirectional relationship between the gut and brain, there has been heightened interest in the role of the gut microbiota as part of this axis. At present most of the evidence for a role of the gut microbiota in brain function, behaviour or mental health originates from animal research or observational studies in humans and is therefore in the very early stages of understanding, though there is gathering evidence of the significant interaction the gut microbiota has in human behaviour, mood, and cognition.

*Animal models*: Animal models for investigating the gut–microbiome–brain axis include germ-free (GF) rodents (bred without a microbiota) and rodents with antibiotic-induced dysbiosis. A meta-analysis of 48 rodent studies investigating the impact of antibiotic-induced dysbiosis reported a 32.7% increase in anxiety and a 40.7% increase in depression-like behaviour in the study populations, respectively (Hayer et al., 2023). There was a significant association between symptoms of negative valence system (including anxiety and depression) and cognitive system (decreased spatial cognition) with antibiotic intake (*p* < 0.05). While the area is promising, the level of heterogeneity between the studies and publication bias makes it difficult to generalise results.

*Human studies*: Observational studies show associations between mood disorders and gut dysbiosis. In a cohort of elderly adults in China, there was a correlation between high concentrations of antibiotics in urine and the presence of depression (Liu et al., 2021b). Using nested case–control studies from a large population‐based UK database, a single antibiotic treatment was associated with a higher risk of depression in all antibiotic groups (Lurie et al., 2015). However, observational studies lack the ability to prove causation. Illness and infection itself (leading to antibiotic use) may promote greater levels of mood disorders.

*Faecal microbiota transplant studies*: Evidence from faecal microbiota transplant (FMT) is emerging to support a role for the microbiota in mood. Transferring faecal samples from the microbiota of humans with depression into GF rats resulted in increased anxiety and reduced interest in pleasurable activities. There was also an association with reduced tryptophan metabolism, the precursor of serotonin (Kelly et al., 2016).

*Irritable bowel syndrome*: IBS is a GI disorder characterised by chronic abdominal pain and altered bowel habits and is one of the most common GI disorders seen by clinicians. People with IBS have a threefold higher increase of anxiety and depression than healthy controls (Zamani et al., 2019), and IBS is described as a disorder of gut–brain interaction (Mearin et al., 2016) with acknowledgement that the gut microbiota likely plays a role.

In summary, the evidence for a role of the gut microbiome in the effects on mood is still in its infancy, but growing as a result of animal and FMT studies, human observational studies, and RCTs in humans investigating the effects of probiotics and prebiotics.

### How might the microbiota signal to the brain?

The routes of communication between gut microbes and the brain are varied and complex, and their understanding is still evolving (Butler et al., 2019). There are a number of pathways and mechanisms between the gut microbiota and the brain, including the vagus nerve and the immune system, besides metabolites and neurotransmitters (Margolis et al., 2021).

Severing the vagus nerve in mice (vagotomy) has helped identify multiple roles for this cranial nerve, including evidence that gut microorganisms can activate the vagus nerve and play a role in mediating the effects on the brain and behaviour (Forsythe et al., 2014). Studies on mice have demonstrated how certain probiotic action desists after a vagotomy. For example, *Lc. rhamnosus* JB-1 has been demonstrated to have an anxiolytic effect in mice. However, mice subjected to vagotomy did not benefit from the reduction in anxiety-like behaviour or reduced corticosterone response to stress, as observed in the control mice also offered the probiotic; demonstrating that signalling is taking place via the vagus nerve (Liu et al., 2021b).

The immune system is beginning to be appreciated as an important factor in mental health. The immune systems in both the gut and brain are connected and can be influenced by the microbiome. As previously discussed by Professor Pot, immune cells possess toll-like receptors that, when activated by the presence of bacteria, initiate a cascade of events resulting in either pro- or anti-inflammatory cytokine production, depending on the microorganisms detected. The presence of pathogens results in the release of pro-inflammatory cytokines and other activated immune cells into the peripheral circulation, which cross the blood barrier and activate microglia (CNS immune cells), potentially causing CNS inflammation (Kasarello et al., 2023). The role of the microbiota in influencing the immune system is supported by research in GF mice that display defects in the maturation and function of microglia, resulting in deficient innate immune responses. The key signalling pathway mediating this influence in mice was found to be bacterial SCFAs (Erny et al., 2015).

SCFA produced by gut microorganisms can communicate both directly and indirectly with the brain and have been reviewed by Berding et al. (2021). SCFA bind onto receptors in the blood–brain barrier indicating the potential for direct communication, with the potential to stimulate neurotransmitter synthesis and influence neuronal behaviour, gene expression, and immune activation (Lan et al., 2024). Butyrate, in particular, has also been shown to enhance the integrity of the blood–brain barrier, protecting it from potential neurotoxic factors which could cause inflammation.

The gut microbiota can also encourage the production of neurotransmitters and their precursors. Tryptophan is the sole precursor of the neurotransmitter serotonin (5-hydroxytryptophan [5-HT]), which is involved in mood regulation. While it is true that 95% of serotonin is produced in the gut (Terry and Margolis, 2017; Appleton, 2018), it is unlikely that this molecule can cross the blood–brain barrier; serotonin produced locally in the gut primarily contributes to gut motility (Berding et al., 2021). However, both precursor molecules tryptophan and 5-HTP can cross the blood–brain barrier. The gut microbiota have been suggested to be able to produce small quantities of tryptophan, the precursor of serotonin, while others metabolise tryptophan into metabolites that can signal directly or indirectly transmitted to the brain (Agus et al., 2018). The gut microbiota also shifts the accessibility of tryptophan to the kynurenine pathway, an alternative metabolic route for tryptophan (Waclawiková and El Aidy, 2018). Recent research in a depressed mouse model has shown reduced levels of tryptophan and 5-HTP in faeces and reduced levels of 5-HT in brain tissue, which are strongly correlated with significantly decreased levels of members of the *Lactobacillus* group in the microbiota (Xie et al., 2023). The mechanisms by which tryptophan metabolism in the gut can affect the brain remains an active area of research.

### The era of psychobiotics, mood, and depression

RCTs investigating the effects of the various “biotics” on the modification of the microbiome and mood are further helping to elucidate this relationship. Psychobiotics is a term commonly used to refer to a live micro-organism that, when ingested in appropriate quantities, has a positive mental health benefit (Butler et al., 2019). It has been suggested that prebiotics should also fall into this class (Yang et al., 2023).

*Trials in healthy subjects*: In a 12-week double-blind RCT of 63 healthy older adults (≥65 years), a probiotic supplement containing *Bifidobacterium* species promoted mental flexibility (ability to switch between different thoughts and actions), improved stress scores versus placebo but did not impact on other self-reported measures of depression (Kim et al., 2021). Brain-derived neurotrophic factor known to be crucial for learning, memory function, and stress was also significantly elevated in the probiotic group after 12 weeks. In a triple-blind RCT including 20 healthy participants, a 4-week probiotic supplement (*Bifidobacterium* and *Lactobacillus* strains) significantly reduced reactivity to sad mood, which was largely accounted for by reduced rumination and less aggressive thoughts as compared to a placebo control group (Steenbergen et al., 2015).

In terms of prebiotics, an RCT using a GOS supplement (5.5 g/day) offered to healthy individuals (n = 45, 18–45 years) for 3 weeks significantly reduced the waking cortisol response and shifted the focus from a negative to a positive imagery in participants versus placebo. There was no effect on self-reported mood, possibly owed to the short duration of the study (Schmidt et al., 2015). A more recent RCT observed the effects of 4-week GOS supplementation (5.5 g/day) in healthy young women (n = 64, 16–24 years), comparing the results between those with high or low anxiety traits. Supplementation with GOS significantly reduced self-reported anxiety scores in women who were highly anxious but not in those with low-level anxiety, versus placebo (Johnstone et al., 2021). A RCT in older adults found that a 7.5-g prebiotic supplement of inulin and FOS improved cognition in older adults over a span of 12 weeks, performing better than the placebo on memory tests typically used to identify early stages of Alzheimer’s disease (Ni Lochlainn et al., 2024).

*Probiotics for depressive symptoms:* The use of psychobiotics is becoming increasingly studied for use in clinical disorders, particularly as an adjunct to current therapies. A meta-analysis demonstrated that probiotics were effective in reducing depressive symptoms when administered in addition to antidepressants but not when used alone (Nikolova et al., 2021). Guidelines issued by the World Federation of Societies of Biological Psychiatry and the Canadian Network for Mood and Anxiety Disorders provisionally recommend the use of probiotic strains (e.g., *Lactobacillus* and *Bifidobacterium* spp.) at doses of between 1 and 10 billion CFUs per day for adjunctive use and are weakly recommended for monotherapy use in major depressive disorder (MDD) (Sarris et al., 2022).

In summary, psychobiotics are an emerging area of research that appears to show a tendency for greater effects in those with mood disorders and with some organisations provisionally recommending probiotics for adjunctive use in MDD. However, additional RCTs are necessary to evaluate their therapeutic benefit in patient populations with psychiatric conditions.

### Practical dietary advice

While there are still gaps in our understanding of the links between specific diets and mood disorders, Dr Leeming indicated that dietary changes to support the gut microbiota include a focus on nutrients, which are key components of a Mediterranean-style diet including fibre, polyphenols, and omega-3 fatty acids.

It is generally recognised that a diet rich in fibre promotes an increase in bacterial diversity and beneficial microorganisms in particular (Berding et al., 2021). In a recent meta-analysis, a higher fibre intake was linked to lower odds of depression (Fatahi et al., 2021), and a separate analysis showed that every 5 g increase in fibre was associated with a 5% lower risk of depression (Saghafian et al., 2023). This is of particular concern as many countries fall short of daily fibre recommendations. In the United Kingdom, the average fibre intake among adults is 19.2 g per day (Public Health England (PHE), 2020) compared to the recommended 30 g per day (Scientific Advisory Committee on Nutrition (SACN), 2015), with similar gaps found in other European countries (Rippin et al., 2017).

Polyphenols are plant metabolites found typically in colourful fruit and vegetables as well as in coffee, chocolate, tea, and red wine. Most polyphenols are not absorbed in the small intestine but are metabolised further down the digestive tract by colonic microflora and emerging data support their effects as prebiotics on the microbiota (Zhou et al., 2020). The inclusion of fermented foods such as kefir, sauerkraut, kimchi, and yoghurt potentially provides beneficial bacteria (Marco et al., 2021). Omega-3 fatty acids contained in foods such as oily fish may also have a prebiotic-like effect (Vijay et al., 2021).

As a note of caution, while diet and lifestyle measures can be used to help those who have sub-clinical depression, the emerging research on food and mood should not delay or desist access to mental healthcare practitioners or prescribed mental health medication, particularly in severe depression.

## Key messages

- A complex bidirectional pathway exists between the gut and brain and also involves the gut microbiota. Most evidence for the role of the gut microbiota in mood and depressive disorders is pre-clinical and observational, with building evidence in control trial settings. However, this is a rapidly escalating field with the potential for a more holistic approach using diet and other lifestyle factors to modulate the gut microbiome as adjunct therapy.
- Depression is a multi-factorial condition, and specific pathways of pathogenesis are individualised and remain elusive, making the research in this area difficult.
- A small number of initial RCTs have shown the benefits of a healthy diet, certain nutrients, and interventions with psychobiotics, but more research is required to unlock the complicated relationships between diet and highly complex organs such as the gut and the brain.

## The microbiome in menopause

Menopause is the final stage of reproductive ageing in women and is defined, retrospectively, when a woman has ceased having a menstrual cycle for 12 months. Symptoms can greatly impact on quality of life and include vasomotor and genitourinary symptoms, mood changes, and sleep disturbances, as well as an increased risk of bone loss and cardiovascular disease (Davis et al., 2015). Dr Brandilyn Peters-Samuelson, Assistant Professor in the Department of Epidemiology and Population Health at the Albert Einstein College of Medicine, USA, discussed changes in the gut microbiome during ageing and menopause, potential health consequences of microbe-hormone interactions, and introduced the concept of the “estrobolome” and implications for menopause symptoms.

### Hormonal changes during menopause

Menopause is the culmination of hypothalamic and ovarian ageing, both of which are influenced by genetic factors, environment, lifestyle, and systemic disease, for example, autoimmune disorders, diabetes, and hypertension. Specific stages of menopausal transition have been defined using the Stages of Reproductive Aging Workshop criteria (Davis et al., 2015). These are based on menstrual, endocrine, and ovarian markers. The menopausal transition is divided into an early stage characterised by fluctuating length of menstrual cycles and variable levels of follicle-stimulating hormone (FSH) and a later stage, defined by amenorrhoea (>60 days) and elevated FSH. Progressing through the stages of menopause, there is a decline in ovarian follicles accompanied by a decline in ovarian hormones (oestradiol, inhibin B, and anti mullerian hormone) and an increase in the gonadotropin-luteinising hormone and FSH. Post menopause, ovarian hormone levels remain low for the remainder of life, and adipose tissue mostly becomes the primary source of remaining oestrogen (Davis et al., 2015).

### Sex- and age-related changes in the microbiota

Menopause-related changes to the gut microbiome need to be considered against the context of age- and sex-related changes in the microbiota. The largest shift in microbiota diversity occurs in early weaning with progression from breast milk to mixed solid foods, and it increases drastically within the first year of life for the infant. Up to approximately 3 years of age is a critical window of opportunity to modulate the gut microbiota composition. After this age, the microbiota is more stable and mature (40%–60% similarity with the adult microbiota) (Koenig et al., 2011; Yatsunenko et al., 2012; Bäckhed et al., 2015). During puberty, changes are observed in the gut microbiota, highlighting the influential role of sex hormones. Research on adolescent mono- and dizygotic twins shows significant differences between the microbiota of male and female twins, not observed in infancy (Yatsunenko et al., 2012). Microbiota diversity continues to increase gradually during adulthood and appears to reach a plateau around 40 years of age. Interestingly, women also have slightly higher microbiota diversity than men in younger adulthood, but not in older adulthood, which may suggest sex differences in microbiota ageing (de la Cuesta-Zuluaga et al., 2019). With increasing age, there is a shift towards a state of uniqueness within each person with the highest uniqueness in long lived individuals which may be related to healthy ageing (Wilmanski et al., 2021).

### Sex hormones and the gut microbiome – a bidirectional relationship?

A bidirectional relationship has been proposed between the gut microbiota and sex hormones. Sex hormones can influence the microbiota and therefore impact microbiota-related mechanisms in health and disease. Similarly, gut microbiota deconjugation of sex hormones can contribute to hormone retention which may, in turn, have an impact on hormone-related mechanisms in health and disease.

Sex hormones originate in various tissues. The ovaries and adipose tissue are the main source of oestrogen in premenopausal and postmenopausal women, respectively. Oestrogens enter the systemic circulation to reach target tissues as well as the liver, where they undergo hepatic conjugation with glucuronide or sulphate groups. Free oestrone and oestradiol are conjugated with glucuronides to form oestrone-3-glucuronide and estrodiol-17-glucuronide. This facilitates their excretion in the bile into the gut, where microbial-derived β-glucuronidase deconjugate and restore oestrogen for reabsorption into the enterohepatic circulation. Similarly, gut microbial sulphatases return oestrone-sulphate and dehydroepiandrosterone-sulphate into the free unconjugated forms oestrone and DHEA; the latter is a precursor hormone which can be converted to oestrogens and androgens. The aggregate of bacterial genes capable of deconjugating and recycling oestrogens is termed the “estrobolome” and may help in the retention of oestrogen in the body, with potential benefits on hormonal symptoms in menopause (Peters et al., 2022). A recent review, however, also discusses the potential carcinogenic role of oestrogen in the light of hormone-susceptible cancers, such as breast, prostate, endometrial, and lung cancers, while also highlighting other factors associated with, for example, oestrogen-driven breast cancer (Al-Shami et al., 2023).

“Estrobolome” enzymes are expressed across a diverse range of microbes and across multiple bacterial phyla, especially species in Firmicutes (now Bacillota) and Bacteroidetes (now Bacteroidota) which express β-glucuronidase enzymes (Pollet et al., 2017). An *in vitro* study showed that different gut bacterial β-glucuronidases had the capacity to deconjugate oestradiol and oestrone glucuronides, to varying degrees, providing a proof-of-principle that human gut microbiota can reactivate oestrogens (Ervin et al., 2019). Similarly, human gut bacterial sulphatases are expressed mostly in the Bacteroidota, followed by the Bacillota phyla. A small cross-sectional study (n = 25 men, n = 7 postmenopausal women, n = 19 premenopausal women) found a significant positive correlation between gut microbial diversity and total urinary oestrogens in postmenopausal women and men, indicating a role of the microbiota in systemic non-ovarian oestrogen levels (Flores et al., 2012). This correlation was not observed in premenopausal women whose levels of oestrogen can vary significantly and where the additional impact of the gut microbiota may be minimal or obscured.

### What happens to the microbiome in menopause?

To determine the effect of oestrogen and menopause status on gut microbiome diversity, premenopausal and postmenopausal women were compared to each other and to men of similar ages in a large prospective cohort study (n = 2300; Peters et al., 2022). Shotgun sequencing of stool samples was carried out to determine gut microbiota taxonomic and functional composition. In the alpha-diversity analysis, premenopausal women had a trend towards a higher gut microbial diversity than postmenopausal women (p = 0.06) and significantly higher diversity compared to both young and old men (p = 0.03) while postmenopausal women did not differ from men. In beta-diversity analysis, postmenopausal women differed significantly in gut microbiome composition from premenopausal women. Furthermore, gut microbiome composition in premenopausal women differed more from younger men than postmenopausal women did from older men. Together, these various comparisons support an effect of female sex hormones on the gut microbiome and that menopause is associated with a gut microbiome that is more similar to men. β-Glucuronidase abundance was significantly higher in premenopausal versus postmenopausal women while sulphatase abundance only differed significantly between premenopausal women compared to men (both young and old). This may suggest that the estrobolome potential, that is, deconjugation activity towards sex steroid hormones, is diminished after menopause due to oestrogen depletion.

The association of sex hormones with the gut microbiome was studied in a sample of 200 postmenopausal women in the USA (Peters et al., 2023a). In terms of alpha diversity, higher levels of serum oestrogens were associated with greater gut microbiome diversity, but this was not consistently observed for other hormones, including androgens and adrenal precursors. In the beta-diversity analysis, serum levels of oestradiol and oestrone were significantly associated with the overall microbiota composition. In this study, a microbiota score (the sum of all gut microbiota species associated with each hormone) was calculated for each of the 15 hormones measured, and a significant positive correlation was found between oestrogen-related microbiota scores and β-glucuronidase abundance. Oestrogen-related species, such as *Alistipes*, were most strongly correlated with β-glucuronidase abundance, which is consistent with prior literature that shows this species to harbour β-glucuronidase genes (Peters et al., 2023a). Thus, in postmenopausal women, higher serum oestrogens are associated with higher gut microbiota diversity, an altered gut microbiome composition, and greater estrobolome potential, suggesting metabolic interaction of the gut microbiota with oestrogens (i.e., “recycling” of oestrogens).

Given that the estrobolome may play a key role in determining oestrogen levels in postmenopausal women, further research is required to determine its impact on health, menopause symptoms, and the influence of diet and lifestyle interventions on its function. For now, a focus on following general healthy eating recommendations including fibre rich foods, can help maintain the diversity of the microbiota while also bringing benefits for other areas related to menopause (e.g., body composition, bone and heart health). Women suffering from symptoms associated with menopause should speak with their healthcare practitioner about options, including hormone replacement therapy (HRT) as well as lifestyle changes, including a healthy diet.

It was highlighted that dietary patterns rich in minimally processed plant foods (i.e, wholegrains, fruits and vegetables, nuts and seeds) and low in refined grains, red or processed meats, and saturated fats are associated with beneficial gut microbiota and also with better cardiometabolic health – which is particularly important in menopause when body composition and cardiometabolic health are affected (Peters et al., 2023b).

### The vaginal microbiota in menopause and the gut–vagina axis

The vaginal microbiome is also of interest during menopause when lower levels of oestrogen cause glycogen levels in vaginal cells to reduce, depleting *Lactobacillus* of their food source and elevating the pH with potential colonisation by pathogens as a consequence (Laniewski and Herbst-Kralovets, 2022). A healthy vaginal microbiota is characterised by high *Lactobacillus* dominance and low diversity. *Lactobacillus* uses glycogen as an energy source and produces a lower pH. The gut microbiome with its “estrobolome” activity could promote oestrogen retention in the circulation, reaching the vagina, and promoting glycogen retention in vaginal cells. This hypothesised connection is referred to as the gut–vagina axis. There are small RCTs which have investigated the effects of probiotics, administered either locally or orally, in both bacterial vaginosis and vulvovaginal candidiasis. These studies have been reviewed by Han and Ren (2021); larger RCTs are needed in this area.

## Key messages

- A bidirectional relationship exists between the gut microbiome and sex hormones. The term “estrobolome” has been coined to refer to the sum of all the gut bacterial genes that are capable of metabolising oestrogens.
- Menopause is associated with lower gut microbiome diversity and lower estrobolome potential versus pre-menopause.
- Serum oestrogen levels are associated with greater gut microbiome diversity and greater estrobolome potential. Metabolic interaction of the gut microbiota with oestrogens may increase their retention in the body with implications for symptom management in menopause as well as reducing menopause-related disease risks related to heart and bone. However, research in this area is in its infancy, and more investigation is needed.
- Women suffering from symptoms associated with menopause should speak with their healthcare practitioner about options including HRT as well as lifestyle changes, including a healthy diet.

## The gut–skin axis

Like the gut, human skin is colonised by a diverse range of bacteria, eukaryotes, and viruses. The role of the skin microbiota in skin disorders, its link with the gut microbiota, and how they are being considered in clinical practice was the subject of the presentation by Natalie Yerlett, dietetic lead for epidermolysis bullosa (EB) and dermatology at Great Ormond Street Hospital, London.

### Comfortable in your own skin microbiome

The skin is the largest organ in the body, and it functions to prevent infection, retain moisture, and regulate body temperature. It is rich in immune cells and heavily colonised by microbial cells. As with the gut, the skin microbiome refers to the community of microorganisms colonising the skin, including structural elements, metabolites, signalling molecules, and the surrounding environmental conditions (Berg et al., 2020). The skin is a harsh environment containing a unique set of microorganisms that can tolerate the salty, dry, nutrient-poor, and acidic conditions, along with fluctuating temperatures and exposure to ultraviolet (UV) radiation (Byrd et al., 2018). Microbial communities on the skin vary between individuals and between their sites on the body. A diverse range of bacterial species can be found, including the genera *Corynebacterium, Staphylococcus, Cutibacterium* (representing some former species of the genus *Propionibacterium), Brevibacterium, Micrococcus*, and *Malassezia* as the main fungal genus (Mahmud et al., 2022). The specific functions of the microbiota living on the skin can be strain-specific; therefore, need careful consideration if therapeutic modulation of the microbiome is to be explored. For example, *Cutibacterium acnes* is a major commensal on skin and plays an important role in skin health, but strains have also been associated with acne (Johnson et al., 2016). Novel species have recently been identified including phages although there is little evidence on how viruses impact skin health (Saheb Kashaf et al., 2022).

The skin microbiota provides protection both directly by competing with pathogens for resources, the release of various chemicals, including antimicrobial factors, and indirectly by interacting with the immune system of the skin, which can differentiate between the commensal bacteria and harmful strains. Thus, the skin microbiota determines the production of pro- or anti-inflammatory cytokines and is also involved in wound healing and strengthening of epithelial tight junctions (De Pessemier et al., 2021); all of which are of relevance to inflammatory skin conditions such as AD. Increasingly, observational studies have shown that the abundance of some pathogenic microbes is associated with skin conditions, but it is difficult to ascertain whether the altered skin microbiome is a cause or consequence of skin disorders (De Pessemier et al., 2021).

### The gut–skin axis in dermatological conditions

The skin and gut share many structural similarities; both are highly innervated and vascularised and have important immune and neuroendocrine functions (De Pessemier et al., 2021). The gut–skin axis describes a bidirectional relationship whereby the gut can influence the condition of the skin and vice versa. Evidence of this interaction is seen in food allergies manifesting in skin symptoms such as urticaria and AD or, in the case of coeliac disease, dermatitis herpetiformis (De Pessemier et al., 2021). Conversely, UV-dependent cutaneous synthesis of vitamin D can influence gut microbial diversity (Bosman et al., 2019), which may affect GI tract integrity. The role of oral prebiotics and probiotics in the amelioration of some skin conditions, such as AD and acne also provide emerging evidence of a gut–skin connection (Mahmud et al., 2022).

### Dysbiosis in skin conditions

While the term dysbiosis is not well defined, it is commonly used to indicate an imbalance of the microbiota correlated with either reduced beneficial strains, over-proliferation of pathogenic strains, and/or reduced overall microbiota variety. Dysbiosis of the gut has been associated with many dermatologic conditions, including acne vulgaris, AD, psoriasis, and rosacea (De Pessemier et al., 2021). What often remains unclear is whether gut dysbiosis influences the dermatological disorder or if the dermatological disorder influences the gut dysbiosis. This cause or consequence question is difficult to solve as many patients with dermatological conditions are frequently prescribed antibiotics that can destroy and/or inhibit the growth of specific microbial groups and/or change the molecular patterns associated with the (skin) microbiome. In these cases, an additional drug factor should be considered. In severe burn patients, an acute systemic inflammatory response spreads throughout the body, and the need to implement antibiotic therapy can quickly result in microbiome dysregulation within the skin, lungs, and gut, which can influence the inflammatory response (Corcione et al., 2020). Disruption of the intestinal barrier in burn patients leads to increased intestinal permeability and the translocation of bacteria and/or endotoxins, causing systemic inflammatory response syndrome. Research is investigating how rehabilitating the gut microbiota may be an efficient adjunct therapeutic strategy in burn patients.

### Acquired skin conditions – AD

AD, often referred to as eczema, is a chronic, relapsing inflammatory skin disease characterised by barrier dysfunction, chronic inflammation, and microbial dysbiosis on the skin (De Pessemier et al., 2021). A range of genetic and environmental factors can increase the risk of developing AD. Specifically, genetic filaggrin gene mutations can predispose individuals to a disrupted skin barrier and dry skin; however, environmental factors such as water hardness, allergens, and irritants can also play a role in the pathogenesis. The severity of AD symptoms varies; therefore, treatment ranges from mild cases necessitating topical treatments only to severe cases requiring hospital admission for wet wrapping or systemic treatments, such as biologics. Treatment often involves antibiotics, which also has implications for the gut microbiome.

*Probiotic research in AD and guidelines for use*: Evidence suggests that individuals displaying gut dysbiosis as infants are much more likely to develop AD (Cheung et al., 2023). Consequently, research has investigated the potential therapeutic use of oral probiotics to modulate GM and its subsequent effects on AD. *Lacticaseibacillus rhamnosus* GG in early infancy has been shown to reduce the incidence of AD in at-risk infants through to the age of 7 years (Wickens et al., 2013), but meta-analyses present a mixed picture. A significant reduction in AD severity scores was observed in those consuming probiotics compared to the control group (25 RCTs; n = 1,599). This effect was observed in children and adults with moderate-to-severe AD but not in infants. Treatment effect was improved with combinations of bacterial species or *Lactobacillus* alone versus *Bifidobacterium* (Kim et al., 2014). In a meta-analysis of eight RCTs conducted on the use of probiotics in infants with AD (n = 741), subgroup analysis revealed that probiotics were protective in moderate-to-severe patients and in preparations containing *Lactobacillus* (Zhao et al., 2018). However, substantial heterogeneity exists with studies differing in duration, type, dosing, and timing of supplementation, diagnostic criteria, and duration of follow-up (Halken et al., 2021), making conclusions difficult. *Lc. rhamnosus, Bifidobacterium animalis* subsp. *lactis*, and *L. acidophilus* probiotics in single- or multi-strain formulations appear to currently hold the most promise for prevention and treatment of AD. The difference between single- and multi-strain products is further discussed by D’Elios et al. (2020).

Guidelines from the American Academy of Paediatrics (Thomas et al., 2010) and the European Academy of Allergy and Clinical Immunology (Halken et al., 2021) do not currently contain recommendations for probiotics to prevent the development of allergic diseases due to the lack of conclusive evidence. However, the World Allergy Organization guidelines conditionally recommend the use of probiotics in pregnant women and breastfeeding women with infants at high risk of allergies and in infants at high risk of developing allergies, with the evidence cited as very low quality (Fiocchi et al., 2015). Safety of probiotics is important, and their use in patients with compromised immune function or serious underlying disease should be considered carefully and restricted to strains with proven safety and efficacy for these specific population groups (World Gastronenterology Organisation (WGO), 2023).

*Practical dietary measures for AD:* Dietary strategies that may offer benefits in high-risk infants include early introduction of allergens during weaning and allergen avoidance only in cases of proven allergic reactions (BDA and BSACI, 2018). Vitamin D levels have been reported to be lower in AD patients of all ages, with symptoms reporting to improve following vitamin D supplementation (Kim et al., 2016). For this reason, vitamin D supplementation as per guidelines for the general population (e.g., 10 μg/day for >4 years age) is recommended (National Health Service (NHS), 2023). While more specific evidence is needed, a varied diet high in fibre and omega-3 fatty acids and containing fermented foods is also thought to be beneficial in ensuring a diverse gut microbiota (Berding et al., 2021; Marco et al., 2021; Vijay et al., 2021), and show promise in helping either prevent or alleviate allergic inflammatory conditions.

### Congenital skin conditions – EB

EB refers to a group of rare inherited skin disorders that cause the skin to become very fragile, leading to painful blistering. In normal healthy skin, the three layers (dermis, basement layer, and epidermis) are held together by “velcro-like” collagen proteins. However, in EB patients, expression of these proteins is absent or patchy, depending on the particular EB type (Yerlett et al., 2022). With friction or tension, skin comes away, leaving large areas of acute dissuasion, similar to burns, alongside regular episodes of inflammation, infection, itchiness, and pain. EB patients often present with high losses of fluid, plasma, and electrolytes from skin exudate, have high nutritional requirements, and often present with significant chronic micronutrient deficiencies.

Elucidation of the role of the skin and gut microbiomes in EB, particularly in reducing inflammation or the need for antibiotics, may pave the way for adjunct therapies intended to improve both skin- and gut-related symptoms as well as quality of life. However, more direct data are clearly needed. One study (n = 28) showed that patients with recessive dystrophic EB (RDEB-S; a faulty gene is inherited from both parents) have significantly reduced alpha diversity in their skin microbiome compared to healthy controls (Reimer-Taschenbrecker et al., 2022). In particular, a predominance of *Staphylococcus aureus* was observed in both wounded and unwounded skin of RDEB-S patients, with higher numbers seen in those with more severe disease.

Dysbiosis has also been associated with EB patients due to frequent antibiotic use and reduced dietary intake. This may consequently result in numerous GI symptoms, including mucosal damage, constipation, protein-losing enteropathy, and bloating (Yerlett et al., 2022). A small study in three children with RDEB-S supplemented with a multi-strain probiotic for 12 weeks found improved stool consistency, reducing the need for chronic macrogol gel use. All three patients reported significant improvements in abdominal pain, bloating, flatulence, and an increase in appetite (Yerlett et al., 2022). As EB is a rare condition, cohort numbers are a challenge, but further research is planned to confirm the benefits of probiotics in this condition. However, improving the quality of life for these patients through alleviation of GI symptoms should not be underestimated.

## Key messages

- The gut–skin axis refers to the bidirectional relationship between the microbiomes of the gut and the skin.
- Dysbiosis of the gut microbiota has been associated with various skin conditions, including AD and EB.
- Some microorganisms on the skin, such as *Cutibacterium acne*, are important in maintaining the normal functioning of the skin microbiota, but they may become linked to skin diseases under specific conditions.
- Continued research on specific probiotic strains and dietary modifications may provide a safe adjunct to existing treatments for dermatologic conditions. *Lc. rhamnosus, Bifidobacterium lactis*, and *L. acidophilus* probiotic strains in single- or multi-strain formulations appear to currently hold promise for the prevention and treatment of AD.
- Understanding the complex relationship between the skin and gut microbiome may help develop new therapeutic intervention strategies in the management of various skin disorders that can potentially improve patient’s quality of life.

## Faecal microbiota transplant: how it works and why sometimes it does not

FMTs are an emerging therapy used in a variety of chronic diseases. The mechanisms through which this apparently miraculous therapy has its benefits are incompletely understood. Dr James Kinross, Senior Lecturer and Consultant Surgeon at Imperial College London explained the current state of the science in this field, how it is being used in practice for chronic functional conditions of the gut, the associated risks and current research priorities in FMT science.

Whilst there is no agreed definition of FMT, it is generally understood to represent the process whereby stools collected from a healthy screened individual (“donor”) are homogenised, filtered, and subsequently delivered into the bowel of an unwell recipient with the aim of re-establishing a more diversified and/or less disturbed microbiota within the gut (Clancy et al., 2021). Its use dates back to ancient times when donor faeces (referred to as “yellow soup”) was used for conditions like traveller’s diarrhoea. However, FMT was launched into western public consciousness in the 1950s when it was used as a treatment for antibiotic-resistant *Clostridioides difficile* (*Cl. difficile*) associated diarrhoea. The last 20 years have seen exponential growth in our understanding of the role of the gut microbiome in health and disease, and there are now over 480 registered clinical trials investigating the use of FMT in a wide variety of disorders, including those not typically associated with the gut, such as Parkinson’s disease, depression, and AD. For an up-to-date overview of ongoing trials, see www.clinicaltrials.gov. Within Europe, a 2019 report showed 31 centres undertaking over 1,874 FMT procedures, with the majority (57%) for the purpose of treating *Cl. difficile* and the remaining 42% for experimental indications such as graft-versus-host disease and antibiotic-associated diarrhoea (Baunwall et al., 2021).

### How is FMT administered in practice?

Water and excess fibre is extracted from donated faecal samples, resulting in a dry mass which is composed of approximately 55% microbes (bacteria, fungi, archaea, viruses) and 24% soluble components (mucus, protein, lipids, SCFAs, colon cells). The FMT sample is homogenised and blended with saline and administered via nasogastric tube, enema, or provided in supplement form. The treatment is regulated in the United Kingdom under the guidance of the Medicines and Healthcare products Regulatory Authority for human medicine regulation. In the United States, the FDA recently approved two procedures for the treatment of recurrent *Cl. difficile* associated diarrhoea (Mullard, 2023).

Notably, as the majority of species in the microbiota are anaerobes, there can be a loss of these important microbes during FMT processing; therefore, adapted, anaerobic techniques are being developed to ensure the preservation of the natural diversity present. The human gut also contains a vast array of viruses, mostly bacteriophages (virus that infect bacteria and *Archaea*), which are increasingly being recognised as an important part of the gut microbiome and, as present in the transplant, warrant further investigation (Shkoporov et al., 2019).

Donors are generally voluntary (in non-commercial organisations) and recommended to be within a certain age range and absent of chronic or metabolic diseases, as confirmed by mandatory screening of donor blood and stool samples (Cammarota et al., 2019). Provision of donor blood and stool samples is strongly recommended during screening for a number of criteria, including the presence of pathogens and multi-drug-resistance bacteria (Mullish et al., 2018). One of the biggest challenges in FMT is accessing and producing sufficient quantities of donor faecal samples. Industrialising the process as a high-throughput intervention could lead to the identification of key strains and their respective effects and greater standardisation of the FMT. However, at present, commercialisation of this process is limited.

FMT is generally considered safe when rigorous screening procedures are in place but there is limited data on the long-term impact and potential risks (Merrick et al., 2020), and for this reason, its therapeutic indications are currently limited. There have been some reported rare side effects of pathogenic organisms being transferred from donor to recipient (Food and Drink Administration (FDA), 2020) as well as reported complications regarding certain routes of administration, for example, risk of regurgitation/aspiration associated with upper GI administration of FMT (Baxter et al., 2015). Professional protocols are becoming increasingly more stringent to manage and prevent such issues (Mullish et al., 2018).

## FMT as a tool to study the microbiome

FMT is also being used as a tool by microbiome scientists to help identify mechanisms of action and to potentially tease out cause-and-effect relationships between microbiome composition and disease. Very often animal models are used, results of which in relation to the human situation should be handled with care. For example, faecal samples collected from children with autism spectrum disorder (ASD) were transplanted into GF mice, leading to ASD-like behaviour in the mice, along with the presence of different microbial community structures and altered tryptophan and serotonin metabolism (Xiao et al., 2021). FMT into mice has been also used to study anastomotic leak, which increases mortality and cancer recurrence in those with colorectal cancer, and to understand which strains may influence immune function and healing following surgery (Hajjar et al., 2023).

### Hair raising questions about mechanisms

The precise way in which FMT works remains largely unknown; however, multiple mechanisms are likely to be involved. At its simplest, it is an ecological intervention to address the loss of diversity (e.g., after antibiotic use) or to outcompete the presence of a pathogen (e.g., *Cl. difficile*). Besides ecological remodelling, potential mechanisms of action may include bioremediation (removal of toxins), re-establishment of gut barrier function, enteroendocrine cell function, hormone bioavailability, immune modulation, and interactions within the ENS (Hanssen et al., 2021). FMT also contains highly biologically active substances such as SCFAs and bile acids, which may also contribute to restoring gut function and barrier health. Mullish et al. (2019) alluded to this multiplicity of mechanisms by hypothesising that in the treatment of *Cl. difficile* infection, the loss of microbiota-derived bile salt hydrolases (BSHs) affects bile metabolism in the gut and that restoring BSHs could be a key mediator of FMTs efficacy in treating *Cl. difficile* infection. In this study, faecal samples from recovered patients demonstrated restored BSH activity and sustained changes in deoxycholic acid excretion. Whilst limited in their scientific rigour, case studies have also reported hair regrowth following FMT in two patients with alopecia universalis treated for recurrent *Cl. difficile* infection, which may be mediated through changes in immune function (Rebello et al., 2017).

### Super donors, super recipients, or best of both?

The phenomenon of “super-donors” has been proposed following the observation that the FMT of some donors results in significantly more successful outcomes than the stool of other donors (El-Salhy et al., 2020). However, without a complete understanding of the mechanisms of FMT, this may not be a true phenomenon, and it has been questioned in at least some indications (Olesen and Gerardin, 2021). FMT efficacy outcomes are complicated and difficult to predict, and the recipient’s existing microbiota as well as the interaction between donor and recipients strains as well as the conditions of the FMT procedure (dose/frequency/route of administration) may also be important. Some research studies suggest that understanding the recipient’s gut microbiota and selecting a donor with a complementary microbiota or metabolic profile could be crucial (Schmidt et al., 2022). Therefore, beyond standard donor screening, matching donors and recipients for microbiota complementarity based on differences in community, species, strains, or even function may improve colonisation success (Schmidt et al., 2022), paving the way for more personalised selection strategies with better prediction of clinical outcomes.

### FMT in clinical practice

In the United Kingdom, current clinical guidelines only recommend FMT for recurrent *Cl. difficile* infection that has not responded to standard therapy, but it is also actively being explored for treating other clinical conditions or as adjunct therapy. The majority of research to date has focused on the use of FMT in gut disorders and was reviewed as part of guidelines from the British Gastroenterology Society and Healthcare Infection Society (Mullish et al., 2018). Meta-analyses have been published on the efficacy of FMT for various GI conditions (some of which are discussed later on), but there is frequently substantial heterogeneity in studies based on the type of processing (aerobic or anaerobic FMT), stool donors (single or pooled), route of administration, and use of antibiotics prior to FMT or bowel lavage. These factors, combined with significant variations in the composition of healthy faecal microbiomes make interpretation of the meta-analysis in relation to the FMT complex.

*Cl-difficile infection:* The National Institute of Clinical Excellence (NICE) in the United Kingdom currently recommends FMT as an option for adults who have been treated more than twice for recurrent *Cl. difficile* infection without success (National Institute of for Health and Care Excellence (NICE), 2022). Clinical trial evidence has shown that FMT treatment is significantly more effective than antibiotics at resolving a *Cl. difficile* infection and is cheaper than almost all other treatment options with antibiotics (National Institute of for Health and Care Excellence (NICE), 2022).

*Inflammatory bowel disease:* FMT has also been considered for the treatment of ulcerative colitis (UC), which can result in changes to the gut microbiota composition, with a loss of *Clostridium* cluster XIVa, butyrate producers, and an overabundance of *Bacteroides*, which may be corrected by FMT (Fuentes et al., 2017). Despite significant sources of heterogeneity in the data as mentioned previously, a meta-analysis appears to favour FMT for UC, based on a reduction in inflammation (El Hage Chehade et al., 2023). In this same meta-analysis, short-term clinical and endoscopic remission were significantly higher in patients who received FMT compared with placebo groups. In terms of Crohn’s disease, a recent meta-analysis (n = 228) showed that 57% of patients (95% CI = 49–64%) experienced some form of clinical remission following FMT, with less heterogeneity in the datasets compared to UC (Zhou et al., 2023).

*Irritable bowel syndrome:* Meta-analyses investigating the impact of FMT on IBS have proved more challenging to interpret, with no clear benefit or mixed results observed. However, many of these studies have not properly phenotyped the IBS subtype and there is significant heterogeneity in the same factors as discussed for other conditions, including the route of administration, which may be of particular significance in IBS. Recent work has shown that administering FMT to the upper GI (via duodenoscopy or nasojejunal tube) may be more effective than colonoscopy in IBS patients (Rokkas and Hold, 2023) but more research is needed.

*Overcoming drug resistance:* FMT is also being explored as an adjunct to medical therapy and in exciting ways to radically change the efficacy of drugs. A relatively new class of drugs, called “checkpoint inhibitors” is used as treatment of advanced metastatic melanoma; however, drug resistance is observed in some patients. The use of FMT has been shown to change the gut microbiota and help overcome drug resistance in some patients (Davar et al., 2021). Thus, the potential exists to use the gut microbiota as a tool to improve the efficacy for certain drugs and to identify potential negative effects of the microbiota on drug metabolism. In parallel, the ability of probiotics to impact drug function, both positively and negatively, is a growing area of research (Merenstein et al., 2023).

### Feeding the FMT

Ensuring a nutritionally adequate dietary intake post-FMT treatment may play a role in sustaining the engraftment, thereby encouraging a successful and effective response following treatment. However, the optimal diet for donors and recipients in FMT still remains to be elucidated, and currently donor screening guidelines do not require a specific diet. In practice, when dietary advice is provided, promotion of government healthy eating guidelines that inherently promote higher fibre diets through emphasis on wholegrains, vegetables, and fruit appear to be most commonly applied (Clancy et al., 2021).

Dietary fibre has been shown to be an important modulator of the gut microbiota and there have been some indications that different types of dietary fibre may be useful. An RCT in severely obese patients with metabolic syndrome receiving oral FMT combined with either high-fermentable or low-fermentable (LF) fibre supplements or placebo supplements only found that patients in the FMT-LF group alone had significant improvements in insulin resistance (HOMA2-IR) at 6 weeks compared to baseline, with no improvement in the other groups (Mocanu et al., 2021).

### Beyond FMT and the gut microbiome – exploring new frontiers

Both viruses and fungi have remained unmeasured in any great detail to date, but they are important components of the gut microbiota. There are a small number of studies investigating faecal virome transplants. Patients with *Cl. difficile* infection who were infused with a donor FMT richer in *Caudovirales* were all cured by FMT, although the mechanisms are still to be defined (Zuo et al., 2018). Specific fungi appear to be important in regulating mucosal and intestinal barrier health and can also be transplanted (Zhang et al., 2021). However, research is in its infancy, and the significance and implications of these alternations in fungal compositions on disease outcomes warrant further investigations.

Microbiota transplants from non-GI area are also being researched. Vaginal microbiota transplants (VMTs) are currently being studied in women suffering from infertility. A single case report in a 30-year-old woman with a history of miscarriages and stillbirths who had a *Lactobacillus*-depleted microbiota had a VMT resulting in an increase in *L. crispatus* and *Lactobacillus jensenii.* This concurred with resolution of vaginal symptoms and a pregnancy 5 months post VMT with consequent live birth (Wrønding et al., 2023).

## Key messages

- FMT is an important experimental tool for understanding the gut microbiome and teasing out cause-and-effect relationships, but it is also a therapeutic tool in its own right. FMT is currently recommended for those with recurrent *Cl. difficile* infection, and research is promising in its use in the treatment of some cases of IBD.
- There is an urgent need to standardise FMT interventions and characterise strains and their functions. New techniques for preserving anaerobes from the donor sample will provide greater diversity in the FMT sample.
- There is a greater focus on understanding both donor and recipient factors that affect successful colonisation post-FMT, and there may be the potential to better match donors to recipients based on a microbiota complementarity.
- Based on what is known about dietary factors and the gut microbiota, ensuring a healthy balanced diet following FMT may support successful engraftment, and patients should be referred to a dietician.
- The long-term risks of FMT are unknown. Thus, therapeutic indications are currently limited and, whilst promising, more research is needed for its use in non-life-threatening conditions, such as IBS.

## Conclusions

The presentations from the 2023 Yakult Science Study Day have shown the gut microbiota as a conductor of health, orchestrating effects that reach beyond the GI tract and ultimately influencing broader health outcomes. A wide range of evidence from pre-clinical animal studies, artificial gut models, through FMT and up to RCTs with dietary interventions, are adding to our understanding of the role of the gut microbiome in modulating health and disease. Recent advances in DNA sequencing technology have increased our understanding of “who is there?” and “what are they doing there?” with the potential to better predict outcomes of microbiota interventions and allow a more personalised approach to modulating the gut microbiome for health. This has also led to greater consideration of the gut microbiome within clinical practice and growing interest in developing microbiome-based therapies. While still in the very early stages, therapies are being researched as either standalone clinical treatments or adjuncts to standard medical therapy in GI-related disorders but also in unexpected areas, such as skin disorders, menopause, depression, and drug efficacy. Diet is an important factor in shaping the gut microbiome and could play a role in either prevention or in “fine tuning” of therapies for microbiota-related diseases with both overall dietary patterns as well as specific pre- and probiotics, and fermented foods showing promise in this area. It is understandable that such fascinating research engages the public imagination; therefore, it is crucial that healthcare professionals and researchers stay abreast of the advances in research, whilst acknowledging current limitations and ensuring that the public is offered evidence-based advice. We have only just begun to explore the vast potential of gut microbiota research and its impact on health. With ongoing scientific advancements, the future holds even more promising discoveries, making it an incredibly exciting field to follow.

## Abbreviations

AD: atopic dermatitis
ASD: autism spectrum disorder (ASD)
*B.*: *Bifidobacterium*
BBB: blood brain barrier
*C.*: *Clostridium*
*Cl.*: *Clostridioides*
CFU: colony forming unit
CNS: central nervous system
DNA: deoxyribonucleic acid
EB: epidermolysis bullosa
ENS: enteric nervous system
FVT: faecal virome transplant
FOS: fructo-oligosaccharides
FMT: faecal microbiota transplant
GI: gastrointestinal
GOS: galacto-oligosaccharides
HMO: human milk oligosaccharides
IBS: irritable bowel syndrome
IBD: inflammatory bowel disease
IL-12: interleukin-12 (similarly IL-10 IL-8)
LAB: lactic acid bacteria
*L.*: *Lactobacillus*
*Lc.*: *Lacticaseibacillus*
LDL-C: low density lipoprotein cholesterol
MDD: major depressive disorder
NK: natural killer
RCT: randomised controlled trial
SCFA: short-chain fatty acid
TNF: tumor necrosis factor
UC: ulcerative colitis
VCAM-1: vascular adhesion cell molecule
VMT: vaginal microbiota transplant.
